# Double Parathyroid Adenoma Presenting as One Mass: A Case Report

**DOI:** 10.15388/Amed.2024.31.1.20

**Published:** 2024-02-27

**Authors:** Rugilė Petruokaitė, Vitalijus Eismontas, Narimantas Evaldas Samalavičius

**Affiliations:** 1 Faculty of Medicine, Vilnius University, Vilnius, Lithuania; 2 Health Research and Innovation Science Center, Faculty of Health Sciences, Klaipėda University, Klaipėda, Lithuania Department of Surgery, Klaipėda University Hospital, Klaipėda, Lithuania

**Keywords:** double parathyroid adenoma, primary hyperparathyroidism, parathyroidectomy, dviguba prieskydinių liaukų adenoma, pirminis hiperparatiroidizmas, paratiroidektomija

## Abstract

**Background and Objectives:**

Primary hyperparathyroidism is a common endocrinological condition, which is usually caused by solitary parathyroid adenoma. The aim of this article is to present a case of double adenoma presenting as one and literature review on this topic.

**Case presentation:**

56 years old female suffering from generalized fatigue, bone pain, unstable body weight and palpitations was diagnosed with primary hyperparathyroidism. Further investigation revealed elevated parathyroid hormone (PTH), ionized and total calcium levels. Imaging showed two adenomas merging into one. Parathyroidectomy was performed and final intraoperative PTH (IOPTH) decreased by 71.6%. Parathyroid adenoma measuring 40 mm x 15 mm x 11 mm and weighing 1 g 483 mg was excised. 7 weeks after surgery patient was feeling well but her PTH level was elevated again.

**Conclusions:**

As far as we know, the double parathyroid adenoma presented in this case report is the largest reported in the Northern Europe and the first one that presents as one mass within the region. Double adenoma increases the risk of recurrent primary hyperparathyroidism (PHPT) and requires more careful and longer follow up after surgery than solitary adenomas. Final IOPTH must decrease by at least 50% within 10 minutes. This case shows that despite final IOPTH drop by more than 50%, after few weeks normocalcemic elevated parathyroid hormone (NCePTH) phenomenon was noticed. This phenomenon does not indicate surgical failure.

## Abstract (it)

**Įvadas ir tikslas:**

Pirminis hiperparatiroidizmas – tai dažna endokrininė būklė, kurios pagrindinė priežastis yra pavienė prieskydinių liaukų adenoma. Šio darbo tikslas – pristatyti klinikinį atvejį apie dvigubą prieskydinių liaukų adenoma, kuri pasireiškia kaip vientisas darinys, ir atlikti literatūros apžvalgą šia tema.

**Atvejo pristatymas:**

56 metų moteriai, besiskundžiančiai bendru nuovargiu, kaulų skausmais, besikeičiančiu kūno svoriu, širdies plakimu, buvo diagnozuotas pirminis hiperparatiroidizmas. Atliekant ištyrimą buvo nustatytas padidėjęs parathormono (PTH), jonizuoto ir bendro kalcio kiekis. Vaizdiniais tyrimais buvo aptiktos dvi adenomos, susiliejančios į vieną. Buvo atlikta paratiroidektomija ir galutinis intraoperacinis PTH (IOPTH) sumažėjo 71,6 proc. Pašalinta prieskydinių liaukų adenoma buvo 40 mm x 15 mm x 11 mm dydžio ir svėrė 1 g 483 mg. Praėjus 7 savaitėms po operacijos pacientė jautėsi gerai, bet tyrimuose PTH lygis vėl buvo pakilęs.

**Išvados:**

Kiek mums žinoma, šiame darbe apibūdinama didžiausia Šiaurės Europoje aprašyta dviguba prieskydinių liaukų adenoma ir pirmoji, pasireiškianti kaip vientisas darinys. Dėl dvigubos adenomos didėja pirminio hiperparatiroidizmo (PHPT) pasikartojimo rizika, todėl šiuo atveju reikalingas atsakingesnis ir ilgesnis stebėjimas po operacijos nei pavienės adenomos atveju. Galutinis IOPTH turi sumažėti bent 50 proc. per 10 minučių. Šis atvejis rodo, kad nepaisant galutinio IOPTH sumažėjimo daugiau nei 50 proc., po kelių savaičių buvo pastebėtas normokalceminis padidėjusio parathormono (NCePTH) fenomenas. Šis fenomenas nereiškia, kad operacija buvo nesėkminga.

## Introduction

Once considered rare, primary hyperparathyroidism (PHPT) is a third most common endocrine disease, that has an incidence of 0.4 to 82 cases per 100 000 persons/year [1–3]. Prevalence of PHPT is higher in perimenopausal and postmenopausal women, for instance, in Scandinavian women of that age it is 2–5% while in elderly men it is 0.73% [1,4]. The main causes of PHPT include solitary adenoma (80%–85%), multi-gland disease (up to 15%), and parathyroid carcinoma (1% or less) [5,6]. Double adenomas (DA) have been reported in up to 12% of patients with primary hyperparathyroidism, where four-gland hyperplasia accounting for the remainder of multi-gland disease (MGD) [7,8]. Double adenoma typically presents as two separate adenomas [8]. In this case, PHPT was caused by double adenoma presenting as one, measuring 40 mm x 15 mm x 11 mm and weighing 1g 483 mg. In contrast, a normal parathyroid gland typically weighs approximately 50–70 mg, and a solitary parathyroid adenoma (PTA) usually is smaller than 2 cm and weighs less than 1 g [9]. As far as we know, this is the largest double parathyroid adenoma reported in the Northern Europe.

Clinical manifestations of PHPT include nephrolithiasis, osteoporosis–osteopenia, pancreatitis, depression, cognitive disorders, and others [2]. However, nowadays it has shifted to a mildly symptomatic phenotype due to more frequent routine blood testing in hospitals and clinics and swift recognition of hypercalcemia [9,10]. The severity of symptoms correlates with the adenoma’s weight and PTH level [2].

PHPT is characterized mainly by elevated parathyroid hormone (PTH) levels, hypercalcemia and hypophosphatemia [3,9]. When it comes to treatment, surgical intervention is curative with cure rates raising from 94 to 99% [7]. The best method is considered to be minimally invasive parathyroidectomy regarding the reduction of surgical complications as well as the cost and length of hospitalization [3].

## Case presentation

**Figure 1:**
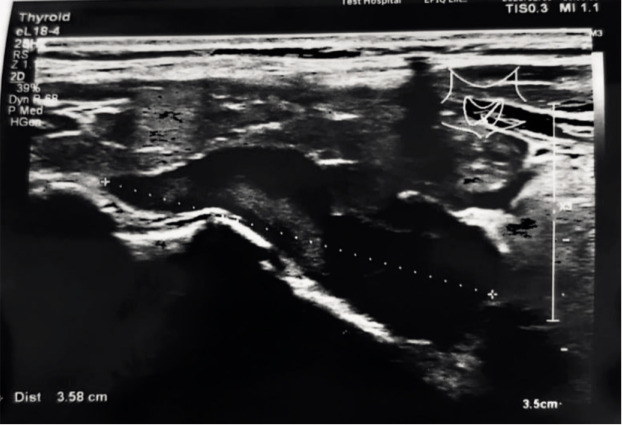
35.8 mm long, hypoechoic nodule with calcification near the left thyroid lobe.

A 56 years old female patient presented to her primary care physician because of generalized fatigue, bone pain, unstable body weight and palpitations. She did not mark discomfort in her neck, dysphagia or hand tremor. Patient was taking levothyroxine (50 mcg once per day) for autoimmune thyroiditis which she was diagnosed with in 2005. In February 2023 neck ultrasonography showed nonhomogenic thyroid with coarsened parenchyma and one large, approximately 35 mm long, hypoechoic nodule with calcification near the left thyroid lobe (Figure 1). She was referred to the outpatient clinic to see endocrinologist for further examination.

**Figure 2:**
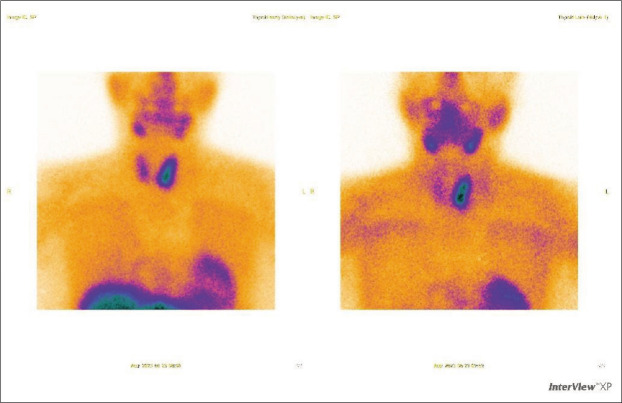
Scintigraphy, early phase. Retention of the radiotracer near the left thyroid gland. Two segments merged into one mass can be identified.

**Figure 3:**
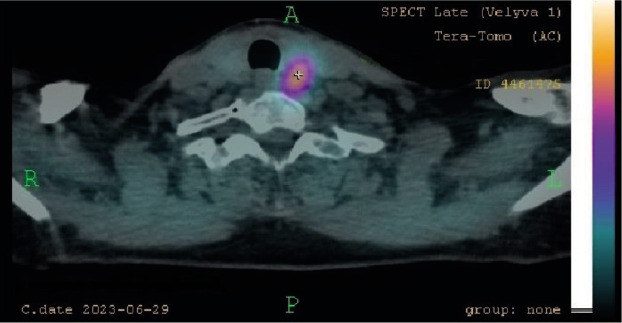
SPECT, late phase. Persistent retention of the radiotracer.

Upon investigation not only elevated parathormone (PTH) level was detected (229.5 pg/ml), but also ionized calcium (1.32 mmol/l) and total serum calcium (3.02 mmol/l). Primary hyperparathyroidism was diagnosed and the patient was scheduled for scintigraphy due to suspicion of parathyroid adenoma. Dual-phase 600 MBq 99mTc-MIBI parathyroid scintigraphy with single-photon emission computed tomography (SPECT/CT) were performed in June 2023. Early and delayed images were analyzed and intensive persistent retention of the radiotracer was visible near the left thyroid gland (Figure 2). Delayed SPECT/CT showed the presence of normal size thyroid with hypodense parathyroid tumor behind the left inferior thyroid lobe and below (Figure 3). This tumor was 33 mm in length and 15 mm in width. It appeared to be two fragments merged into one mass and these fragments might have been superior and inferior parathyroid glands. These findings explained primary hyperparathyroidism and were compatible with the nodule found in ultrasound exam in February. Thus, large left parathyroid adenoma diagnosis was confirmed.

**Figure 4:**
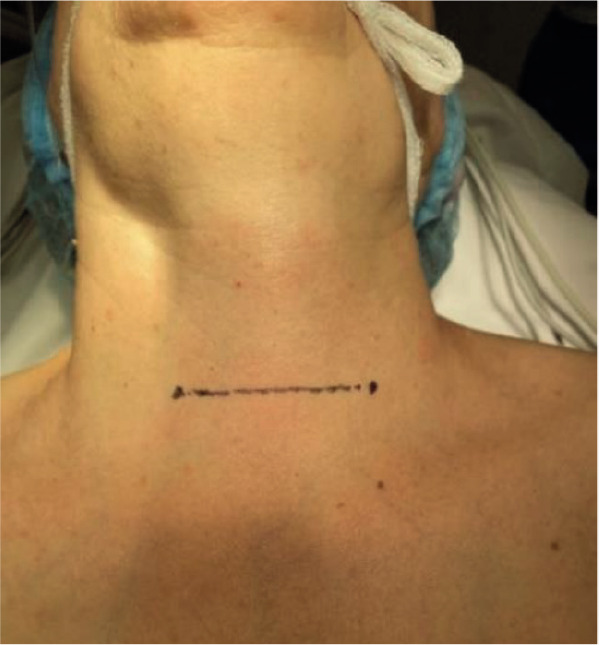
Patient’s neck is normal size before surgery.

In July patient was recommended to visit surgeon and discuss surgical treatment plan. It was confirmed to perform open left parathyroidectomy in Klaipeda university hospital. During physical examination before surgery neck size and patient’s voice were normal (Figure 4).

**Figure 5:**
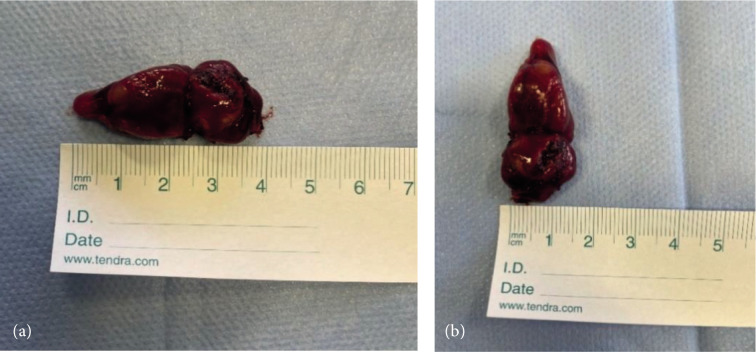
Excised double parathyroid adenoma, which has got two fragments. In total it is 40 mm long (a) and 15 mm wide (b).

During surgery parathyroid adenoma was recognized and excised (Figure 5). Macroscopically, it was ovoid in shape, measuring 40x15x11 mm and weighing 1 g 483 mg. In the picture it can be seen that this adenoma has two parts as it was seen in ultrasonography and scintigraphy. The other parathyroid glands appeared normal.

**Figure 6:**
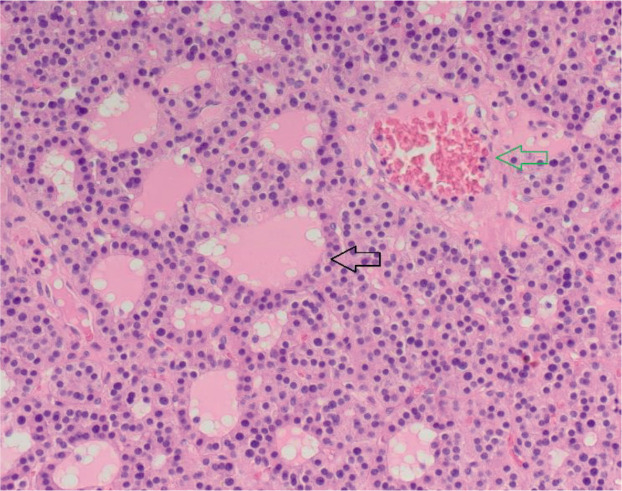
Histopathological view of the excised adenoma. Black arrow marks follicles filled with eosinophilic colloid material and vacuolization on its periphery, that shows active resorption of tumor produced product. Green arrow points to large capillary with intralumenal erythrocytes.

PTH level was evaluated right before skin cut (240.6 pg/ml) and 10 minutes after parathyroidectomy (68.33 pg/ml). As a result of surgery, PTH level decreased almost 4 times. Later the specimen was sent to the pathology department and histopathological examination confirmed clinical suspect of parathyroid adenoma (Figure 6). Microscopic examination revealed chief cells arranged in nests, acinus, trabecular structures with granulated eosinophilic cytoplasm and minimal nuclear polymorphism, without necrosis or mitoses. Peripheral vacuolization of colloid filled follicles showed active resorption of tumor produced product. Neoplasm was demarcated with a thin capsule but there were no other soft tissues, so it was impossible to evaluate invasion.

Postoperative period went smoothly without any events and the patient was discharged the same day. Patient regularly visits primary care physician and endocrinologist for follow up. 7 weeks after surgery patient’s PTH level remained elevated (117.09 pg/ml), but eucalcemia was reached (ionized calcium was 1.076 mmol/l, total calcium was 2.42 mmol/l). Furthermore her 25-hydroxyvitamin D was slightly decreased (29.21 ng/ml). Moreover, osteoporosis was confirmed with DEXA scan. Nevertheless, she is feeling well and has no complaints.

## Discussion

Primary hyperparathyroidism (PHPT) is a prevalent endocrine disorder, affecting approximately 1% of adults in the general population [2,7]. It can be caused by parathyroid gland hyperplasia, multi-gland disease, parathyroid carcinoma, but the most common cause is parathyroid adenoma [2,5,6]. Even though parathyroid carcinoma is the least common cause of PHPT, it always should be considered in the differential diagnosis of PHPT [2,3].

In the presented case the cause of PHPT was double parathyroid adenoma (DA). Some investigators have doubts that double adenomas exist as distinct pathological entity and suggest that they represent cases of asymmetric or asynchronous parathyroid hyperplasia [8,11]. This opinion occurs due to some studies, which show that persistent and recurrent disease is more common among patients with DA than those with single adenoma [8,11]. On the other hand, in some studies, there were no significant difference, so existence of double adenomas was confirmed [7]. Due to uncertainty about relationship between double adenoma and persistent or recurrent disease, it is important to provide these patients close follow-up with serum calcium level after surgery [7,11].

Hypersecretion of parathyroid hormone (PTH) is the key pathophysiological mechanism of hyperparathyroidism [2,12]. It occurs due to abnormal parathyroid cells functioning, because of their lost sensitivity to calcium concentration [12]. PTH inhibits osteoblasts and stimulates osteoclasts activity and therefore calcium is released from bone cells [2,12,13]. Moreover, PTH stimulates calcium reabsorption in kidneys, affects vitamin D renal metabolism and increases calcium absorption from intestines [1,2,13]. These mechanisms are the fundamental reason why hyperparathyroidism leads to hypercalcemia, which usually affects bones and kidneys and causes symptoms, such as fatigue, bone pain, generalized pain, osteoporosis–osteopenia and fractures, recurrent kidney stones, nausea, vomiting, constipation, impaired mentation, neuropsychiatric symptoms and others [1,2,9,10,12]. These symptoms are usually related to severe hypercalcemia, however, when hypercalcemia is mild to moderate, patients can have less symptoms or none [1,10,12,13]. Thus, early recognition of hypercalcemia and elimination of its cause is crucial. In this case, patient presented with bone pain and generalized fatigue that are typical symptoms of PHPT. Her kidneys were not affected by hypercalcemia (her creatinine was 59.45 µmol/l and glomerular filtration rate (GFR) was 96.35 ml/min/1.73m2).

Usually, patients are asymptomatic and diagnosis is made when elevated calcium and PTH levels are detected on routine testing [9,10,12]. If they have symptoms and primary hyperparathyroidism is suspected, investigation begins with serum calcium and PTH levels and progress to imaging in order to localize the cause of this condition [9]. Some authors found that higher preoperative PTH level is related to larger adenomas and to higher risk of recurrence [2,7,14–16]. Considering given information, it is important to know exact PTH level before parathyroidectomy, as it is related to expected size of adenoma and outcomes afterwards. As for this case, diagnostic studies started with an ultrasound, where few nodules were detected. Then patient was referred to the endocrinologist, who took biochemical markers and found out that woman had elevated PTH (229,5 pg/ml) and moderate hypercalcemia (total calcium – 3,02 mmol/l and ionized calcium – 1,32 mmol/l), which confirmed PHPT [13]. Parathyroid adenomas can be detected on neck ultrasonography, magnetic resonance imaging (MRI), computed tomography (CT), 99mTc-MIBI single photon emission CT or scintigraphy [10,12]. The parathyroid glands usually are not seen on ultrasound, but parathyroid hyperplasia or adenoma can be seen as hypoechoic nodule behind the thyroid with vascularization demonstrated by Doppler [12]. The combination of neck ultrasound and 99mTc-MIBI scan is the gold standard for preoperative localization of parathyroid glands, which is essential information for surgery planning [3,9]. Localization of the parathyroid tumor is predicted correctly in 97% of cases when using these methods together [3]. Because of that, patient of this case was sent to do scintigraphy to evaluate function of her thyroid and parathyroid glands.

Parathyroidectomy is curative treatment of parathyroid adenoma [3,7]. It reduces complications (mainly hypoparathyroidism), hospitalization time, and is the most cost-effective method [3,7,12]. It is recommended for young patients, also for those with high serum calcium, osteoporosis, renal calcifications, or the presence of symptoms [3]. Patients who do not meet criteria for parathyroidectomy must be followed and evaluated annually [12]. Even though there are some studies, that show possibility of persistent PHPT or its recurrence, surgery has high cure rates – success is reached from 94 to 99% of cases [7,11,12,15]. Success usually is defined as normalization of serum calcium 6 months after parathyroidectomy [16]. On the other hand, there is a group of patients with elevated post-surgical PTH but normal serum calcium level, while recurrence of PHPT is suspected only when both, PTH and serum calcium levels, are elevated [16,17]. This phenomenon is called normocalcemic elevated parathyroid hormone (NCePTH) and is noticed in 8–40% of parathyroidectomy cases [17]. It is important for medical personnel and patients to understand that this phenomenon does not indicate operative failure, thus no additional surgical explorations are needed [16,17]. Studies show that over time elevated PTH normalizes, however, in long-term follow up it tends to fluctuate [16]. Factors associated with normocalcemic PTH elevation are higher preoperative PTH and/or preoperative serum calcium level, lower preoperative vitamin D concentration, greater adenoma weight, older age, low creatinine clearance, higher 10-min IOPTH, changes in calcium-sensing receptors in the remaining glands, lower peripheral sensitivity to PTH [16,17]. Some studies have shown improvements in normocalcemic elevated PTH with the use of oral calcium and vitamin D supplements [16]. Multi-gland disease is also a risk factor for intraoperative failure, persistent PHPT, and recurrence [7].

Current criteria are insufficient to fully separate DA from asymmetric or asynchronous hyperplasia [7,11]. While removal of true double adenoma usually cures PHPT with significant improvement of complications, elevated risk of persistence or recurrence remains in cases of asymmetric or asynchronous hyperplasia [7,10]. To avoid failures, it is important to monitor IOPTH (intraoperative parathyroid hormone) carefully during surgery [7]. The goal is PTH drop by 50% within 10 minutes after parathyroid gland excision [7,11,12]. Nevertheless, some authors recommend that IOPTH should be decreased not only by 50%, but also to a normal range in order to avoid missing multi-gland disease [18]. There is increased risk for cure failure, when PTH remains elevated, thus patients with a final IOPTH level in the interval 41–65 pg/mL must get at least 6 months long follow-up for recurrence [18]. Furthermore, it is very important to differentiate between PTA and parathyroid carcinoma but it can be challenging in preoperative period [3]. For this reason, surgical specimens should be evaluated by pathologists [3]. Preoperative fine-needle biopsy is not recommended due to the increased risk of dissemination [19].

Female patient in this case had high total calcium and suffered from bone pain, so it was decided to perform an open parathyroidectomy. Specimen was sent for histopathological examination, which confirmed parathyroid adenoma. Final IOPTH was outside the normal range (68.33 pg/ml) and 7 weeks after surgery patient’s PTH level elevated to 117.09 pg/ml, but serum calcium and total calcium levels remained normal. It might be usual PTH fluctuation in postoperative period. However, it can also be due to double adenoma, hyperplasia of remaining parathyroid glands, decreased vitamin D concentration, higher 10-min IOPTH, osteoporosis [7,16]. Calcium and vitamin D supplements should be suggested for this patient. Close follow up for at least 6 months and regular evaluation of PTH and calcium levels, vitamin D concentration and renal function are essential in this case, because this female has few risk factors for persistent or recurrent PHPT (higher final IOPTH and PTH after 7 weeks, multi-gland disease) [7,16,17].

## Conclusions

As far as we know, the double parathyroid adenoma presented in this case report is the largest reported in the Northern Europe and the first one that presents as one mass within the region. Double adenoma elevates the risk of recurrent primary hyperparathyroidism (PHPT) and requires more careful and longer follow up after surgery than solitary adenomas. Because of that, it is important to perform a proper investigation and find the real cause of primary hyperparathyroidism. Investigation begins with serum calcium and PTH levels and progress to imaging in order to localize the cause of PHPT. Even though surgery is the only curative treatment, it is not recommended for all patients. When parathyroid adenoma is excised, IOPTH must decrease by at least 50% within 10 minutes. This case shows that despite IOPTH drop by 71.6%, after 7 weeks PTH level elevated again but eucalcemia remained. This phenomenon is called normocalcemic elevated parathyroid hormone (NCePTH) and it does not indicate surgical failure, therefore no further surgical explorations are recommended. It might be due to normal PTH level fluctuation but also remaining parathyroid gland hyperplasia is possible, thus close follow up for at least 6 months is essential.

## Data Availability

The data presented in this study are available from the corresponding author.
